# Pushing the resolution limit of coherent diffractive imaging

**DOI:** 10.1038/s41377-025-01963-2

**Published:** 2025-08-28

**Authors:** Li Liu, Jinxiang Du, Bailin Zhuang, Ming Gong, Jiamin Liu, Honggang Gu, Shiyuan Liu

**Affiliations:** 1 School of Mechanical Science and Engineering, Huazhong University of Science and Technology, Wuhan, Hubei China; 2 School of Optical and Electronic Information, Huazhong University of Science and Technology, Wuhan, Hubei China; 3 Optics Valley Laboratory, Wuhan, Hubei China; 4 Guangdong HUST Industrial Technology Research Institute, Guangdong Provincial Key Laboratory of Manufacturing Equipment Digitization, Dongguan, Guangdong China

**Keywords:** Imaging and sensing, Sub-wavelength optics, Displays, Microscopy

## Abstract

Coherent diffractive imaging (CDI), with its lensless geometry and theoretically perfect transfer function, is considered as one of the most promising paradigms to achieve the Abbe resolution limit. However, recent advances on pushing the resolution limit in high-numerical-aperture (NA) CDIs has thus far been challenging. Here, we report a nearly 0.9NA CDI with an optimized imaging factor (*k* = 0.501), pushing the Abbe resolution diffraction limit for the first time in ultra-high-NA scenarios. Leveraging this the ultra-high NA and the Abbe-limit *k*-factor, we demonstrate a record-high imaging resolution of 0.57 *λ* for CDIs. Our approach builds upon a novel computational framework termed ‘rigorous Fraunhofer diffraction’ that eliminates the Ewald sphere effect in CDIs, particularly for high NAs. Our framework transforms the general challenge of high-NA, resolution-limited CDIs from relying on approximate and complicated geometric corrections to a solvable problem through rigorous model-based computation.

## Abstract (web summary)

Computational framework of RFD-based CDIs.

## Graphical abstract

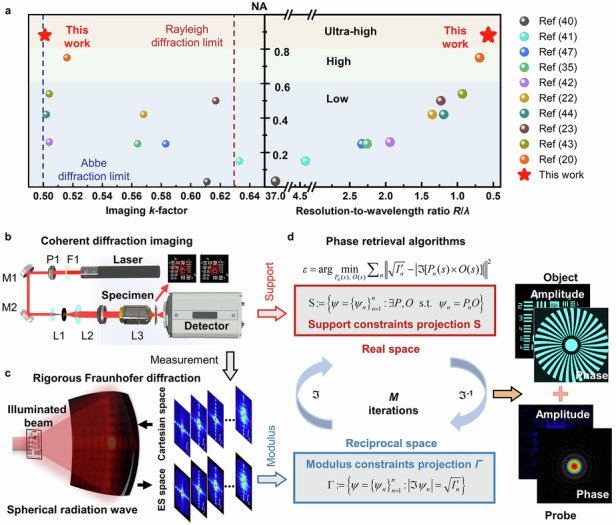

## Introduction

In practical optical imaging systems, the lateral resolution is often defined by $R=k\frac{\lambda }{NA}$, where *λ* represents the illumination wavelength, *NA* denotes the numerical aperture of the imaging system, and *k* is the imaging factor related to multiple variables including illumination conditions, signal distortions, and sample characteristics^1–4^. In the Abbe resolution limit, the imaging factor is 0.5, specifying the theoretical limit of the ultimate resolvable distance of a perfect imaging system. Due to unavoidable diffraction effects of light waves, the imaging factor *k* increases to 0.61, known as the Rayleigh diffraction limit, which provides a practical frontier that cannot be overcome with a conventional imaging system^5^. In pursuit of higher resolution, apart from focusing on shorter wavelength or higher NA^6–9^, more recently, there has been a growing emphasis on reducing the *k*-factor by optimizing the imaging process to approach the ideal transfer function or even break the Abbe resolution limit in diverse fields, such as microscopy, lithography, astronomy, X-ray crystallography, quantum optics, and super-resolution optics^10–15^.

**Fig. 1:**
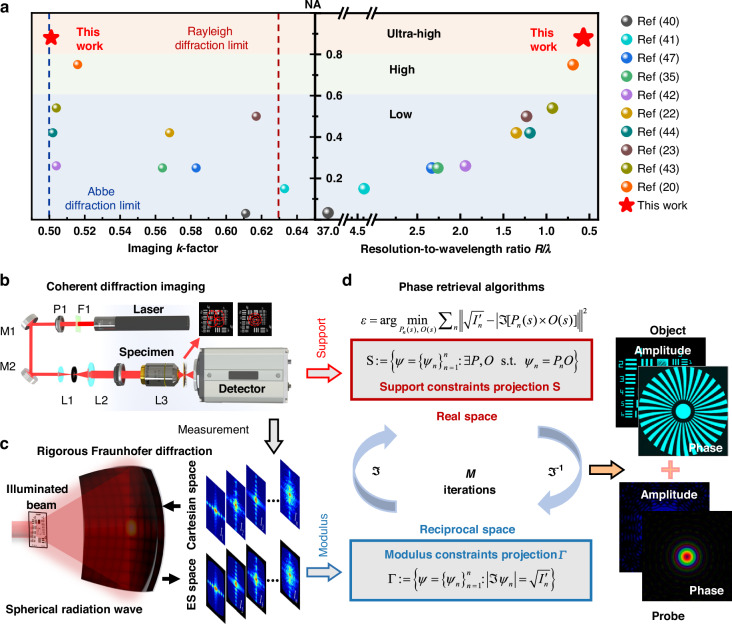
**Computational framework for RFD-based CDIs**. **a** Comparison of the state-of-the-art CDIs that approach the diffraction-limited resolution. **b** CDIs with various real-space encodings. **c** Rigorous Fraunhofer diffraction. **d** Phase retrieval algorithms

Coherent diffractive imaging (CDI), featuring a lensless geometry and a theoretically perfect transfer function, is considered to be the most promising imaging paradigm for achieving the Abbe resolution limit. Combined with innovative architectures and efficient algorithms, CDI continues to refresh the physical bandwidth limits of the imaging system^16–18^. With these efforts, considerable critical issues, such as aberration^19,20^, vignetting effect^21^, depth-of-field and field-of-view constraints^22–24^, coherence degradation^25–27^, as well as system errors^28–31^ and artifacts^32–34^, have indeed been addressed or significantly mitigated, enabling CDIs to easily approach or surpass the Rayleigh diffraction limit (*k*_Rayleigh_ = 0.61)^35–41^. Even more, certain advances have successfully pushed the imaging factor approaching the Abbe limit (*k*_Abbe_ = 0.5)^42–44^. However, reported CDIs that approach the Abbe limited resolution are all achieved under low NA ( < 0.6). In high NA ( ≥ 0.6) scenarios^45^, the imaging factor falls short of the Abbe limit of 0.5^20,22,36–39^. Surprisingly enough, up to now, no lensless CDI with an ultra-high NA ( > 0.8) has been reported. Limited by the low NA and poor imaging process, the lateral resolution achieved so far has been restricted to 0.69 *λ* for real-world objects via CDIs^20^. Figure 1a provides a comprehensive overview of the state-of-the-art CDIs that approach the diffraction-limited resolution. In Fig. 1a, the system numerical aperture NA and the practical imaging *k*-factor, as well as the resolution-to-wavelength ratio *R*/*λ* are demonstrated and compared in state-of-the-art CDIs.

In this paper, we demonstrate the theoretical possibility of achieving perfect transfer functions in CDIs with various NAs and successfully push the imaging resolution to the Abbe diffraction limit. Particularly in lensless ptychography, for the first time, we report an ultra-high NA of nearly 0.9 in the imaging system. In such ultra-high NA setup, we have optimized the *k*-factor to 0.501, thereby succeeding in pushing the Abbe resolution limit. Benefiting from the ultra-high NA and the Abbe-limit *k*-factor, we have achieved an imaging resolution of 0.57 *λ* for CDI, which represents the highest level to the best of our knowledge. The core innovation is that we proposed a computational framework called ‘rigorous Fraunhofer diffraction (RFD)’, in which the rigorous Taylor expansion is adopted instead of the approximate McLaughlin-type binomial expansion commonly used in CDIs. It is the first time that the high-NA, resolution-limit CDI problem is solved once and for all in the Ewald sphere (ES) space by rigorous model-based computation. This computational framework thus prevents the approximate gap between the spherical wave mapped from Cartesian diffraction patterns and the parabolic wave solved by the forward-backward diffraction propagation model in the geometric corrections (GC) method^46–50^. It also overcomes the limitations on propagation distance in the scalable angular spectrum propagation models^51,52^. More importantly, note that the RFD computational framework is essentially rooted in rigorous scalar diffraction theory, and it remains applicable in low NA scenarios. Moreover, it can be applied to extensive realms involving forward and backward diffraction propagations, optical spatial imaging, phase retrievals of inverse problem, wavefield communications and sensing, as well as optical calculations and encryption. Of course, the wave fields are not restricted to electromagnetic fields, and applications in related fields dealing with wave propagation such as acoustics, seismology and wave mechanics can also be expected to be plentiful.

## Results

### Computational framework for RFD-based CDIs

The proposed computational framework for RFD-based CDIs is shown in Fig. 1b–d. In such lensless geometries, the measured signal is typically a diffractogram (or a sequence of such). The measured diffraction pattern in reciprocal space, along with various encodings in real space, combined with rigorous forward-backward diffraction propagation models, i.e., the proposed RFD, are frequently utilized to reconstruct iteratively the complex field by the phase retrieval algorithm with diffraction-limited resolution, resulting in an image of the specimen of interest or the wavefront of the illumination.

**Fig. 2:**
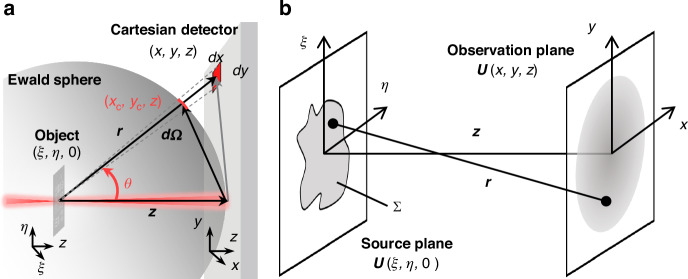
**Free-space optical field propagation**. **a** the Ewald sphere effect. **b** the propagation geometry of the Rayleigh-Sommerfeld diffraction. Here, *U*(*ξ*, *η*, 0) and *U*(*x*, *y*, *z*) represent the optical fields of source plane and observation plane, respectively; *ξ* and *η* are coordinate variables in the source plane; *x*, *y*, *z* are coordinate variables in the observation plane of the Cartesian space and *x*_*c*_, *y*_*c*_, and *z* are coordinate variables in the real ES space; *dx* and *dy* are pixel size in the Cartesian space. ∑ is the optical field region of the source plane. *z* and *r* represent the optical field propagation distance between the source plane and the observation plane and the relative distance of the coordinate positions in the source plane and the observation plane, respectively. *θ* represents the diffraction angle. *dΩ* is the pixel solid angle

Conventional Fraunhofer diffraction (CFD) solver for CDIs only achieves an exact numerical accuracy in the far-field region. In the high-NA scenario of the near-field region, there are inherent diffraction distortions between the Cartesian plane detector and the real spherical radiation wave, known as the ES effect in Fig. 2a, which are usually not negligible. The GC method provides a coordinate mapping from the Cartesian plane detector to spherical space in measured diffraction patterns of reciprocal space, but the forward-backward diffraction propagation model for CDIs (near-field Fresnel diffraction) can only solve for parabolic diffraction distributions. This misalignment, i.e., the spherical space of measured diffraction patterns and the parabolic space of the diffraction propagation model, allows the ES effect to be mitigated but not completely eliminated (more detailed in Supplementary Fig. S1). To eliminate the ES effect once and for all, we innovate to solve this problem by model-based computation in diffraction propagation, rather than the approximate geometric corrections. In the forward-backward diffraction propagation model, starting from the Rayleigh-Sommerfeld (RS) diffraction integral of the free-space optical field propagation once again in Fig. 2b, we employ the rigorous Taylor expansion instead of the approximate McLaughlin-type expansion in the distance term *r* to eliminate the paraxial approximation and the binomial approximation in the diffraction propagation. An entirely novel RFD propagation model is deduced as (more detailed in Materials and Methods).

1 $$\begin{array}{c}U^{\prime} ({x}_{{\rm{c}}},{y}_{c},z)=\frac{z}{i\lambda ({x}^{2}+{y}^{2}+{z}^{2})}\exp (ik\sqrt{{x}^{2}+{y}^{2}+{z}^{2}}){\iint }_{\varSigma }U(\xi ,\eta ,0)\times \\ \exp \left[-\frac{i2\pi }{\lambda z}\left(\frac{x}{\sqrt{1+{(x/z)}^{2}+{(y/z)}^{2}}}\xi +\frac{y}{\sqrt{1+{(x/z)}^{2}+{(y/z)}^{2}}}\eta \right)\right]d\xi d\eta \end{array}$$

which gives the rigorous propagation model gaps in curvature distortion and intensity normalization diffraction fields between the real ES space and the Cartesian plane detector. Therefore, in the proposed computational framework, the ES effect elimination requires only one model-based computation to project a diffractogram (or a sequence of such) of the various encodings from the Cartesian space to the ES space before the inverse problem inversion, which does not impose any additional computation burden on phase retrieval for CDIs. Here, *i* represents the imaginary number, *k* is wave number and *λ* is the wavelength.

Once diffracted signals in real physical spherical space are accurately calculated, the ultimate goal of the phase retrieval inverse problem becomes the pursuit of the minimum Euclidean norm *ε* between the RFD diffractograms and the reconstructed probe-object in the ES space.

2 $${\varepsilon }={\text{arg}}\mathop{{{\min }}}\limits_{{{P}}_{{n}}({s}),{\text{}{O}}({s})}{\sum }_{{n}}{\left|\left| \sqrt{{{I}{{^\prime} }}_{{n}}({{u}}_{c})}-|\Im [{{P}}_{{n}}({s})\times {O}({s})]|\right|\right| }^{2}$$

where, *s* and *u*_*c*_ are spatial coordinates in the real domain and frequency domains, respectively. *n* is the scanning number in the support constraint. *P*_*n*_(*s*) are the overlapped probes on the sample surface in the real space and *I’*_*n*_ (*u*_*c*_) are the multi-frame intensities by RFD in the reciprocal space. *O*(*s*) represents the specimen to be measured called “object.” ℑ is the rigorous diffraction propagation model. By introducing the exit-wave auxiliary variables *ψ*_*n*_(*s*), the Euclidean norm of the phase retrieval inverse problem in the real space and the reciprocal space has been transformed into various optimization problems (more detail in Supplementary Note 2):

3 $$\begin{array}{l}S:=\left\{{\psi }={\{{{\psi }}_{{n}}({s})\}}_{n=1}^{n}:\exists {P},{O}\,{\rm{s}}{\rm{.t}}{\rm{.}}{{\psi }}_{{n}}({s})={{P}}_{{n}}({s}){O}({s})\right\};\\ \varGamma :=\left\{{\psi }={\{{{\psi }}_{{n}}({s})\}}_{n=1}^{n}:|\Im {{\psi }}_{{n}}({s})|=\sqrt{{{I}{{^\prime} }}_{{n}}({u}_{c})}\right\}\end{array}$$

where, *S* and *Γ* are the real-space support constraint projection and the reciprocal-space modulus constraint projection, respectively. After *M* loop iterations, the amplitude-phase complex fields of the object and the probe are iteratively reconstructed from the phase retrieval inverse problem.

### Pushing the resolution limit of in-line holographic CDI

**Fig. 3:**
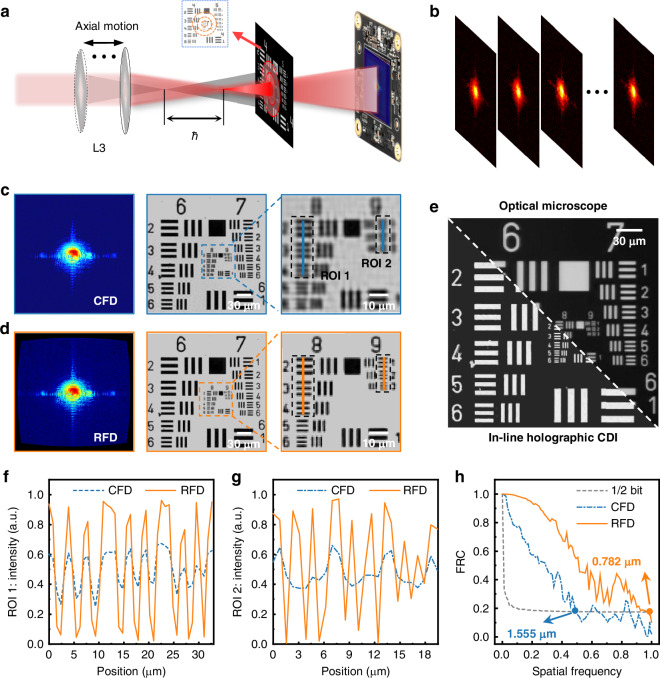
**Experimental results of the in-line holographic CDI**. **a** Optical schematic of the in-line holographic CDI; **b** Experimental raw measured diffraction fields; **c**, **d** Imaging results by the CFD and the RFD. **e** Imaging results of the in-line holographic CDI and optical microscope. **f**, **g** the line traces of the different ROIs. **h** the FRC by the CFD and the RFD

Figure 3a, b shows the optical schematic and the experimental raw measure diffraction fields in the in-line holographic CDI, respectively. After rigorous model-based computation by the RFD, the original hologram and the corresponding result are shown in Fig. 3c, d. In fact, in the real physical scenario, it is not only the lateral misalignments that need to be considered^33,34^, but the specimen-detector diffraction distance is more important since its uncertainty will lead to the scaling of the reconstruction pixel size, i.e., the imaging resolution, and further introduce spatially relevant artefacts. Therefore, the direct holographic reconstruction is extremely hazardous if the diffraction distance is not well calibrated. In order to achieve higher reconstruction quality, an adaptive total variation autofocusing strategy was applied before the holographic reconstruction instead of the classical L_2_ norm (more detailed in Supplementary Note 3). Compared with the L_2_-norm autofocusing strategy, the adaptive L_p_-norm total variation has the faster convergence speed and higher convergence accuracy, and significantly eliminates reconstruction artefacts in Supplementary Fig. S3. After the error calibration of the diffraction distance, the holography algorithm based on the alternating projection^23,53^ in Supplementary Fig. S2 was performed to loop iteration 1000 times on the Intel i7-12700F with NVIDIA RTX 3060 Ti. Regardless of the holographic reconstruction by the CFD or the RFD, we both adopted an adaptive step size strategy to increase the convergence robustness in the experiment, and the iteration parameters were set to be the same to avoid crosstalk from other factors. In the reconstruction results of Fig. 3d, the linewidth features of the Group 9/Element 2 (resolution: ~0.780 μm) can be clearly resolved after the ES effect elimination, while the direct reconstruction by the CFD can only resolve the linewidth features of Group 8/Element 3 (resolution: ~1.550 μm) in Fig. 3c, delivering a resolution enhancement of approximately 2 folds. In the meanwhile, in reconstruction contrast and uniformity, there are also notable improvements of the RFD in Fig. 3d. Figure 3e shows the comparison results between the in-line holographic CDI imaging (equivalent to 0.406 NA in the current configuration) and the optical microscope (BX53MTRF-S, 20×/0.5NA, Olympus). Compared with the truth image measured by the optical microscope, the comparison results perfectly validate the rigorous numerical accuracy of the proposed RFD for the ES effect elimination, except for the misalignment in the diagonal details, which results from the different inherent resolution of the imaging systems.

To eliminate the uncertainty from the qualitative observations, the line trace^23^ and the spatial frequency^42,44^ were employed to quantitatively evaluate the in-line holographic CDI imaging quality. In the line traces of the different ROIs in Fig. 3f, g, regardless of in the resolution or the contrast, there is a significant improvement in the holographic reconstruction by the RFD. It can be clearly seen that the holographic imaging resolution by the RFD reaches Group 9/Element 2, while the result by the CFD without the ES effect elimination is still limited to Group 8/Element 3. Even in the linewidth scales (Group 8/Element 1) resolved by both reconstructions, the holographic reconstruction of the RFD shows significantly sharper steps and better homogeneity. In the Fourier ring correlation (FRC^54^) of Fig. 3h, the holographic reconstruction result of eliminating the ES effect quantitatively demonstrate an approximately twofold enhancement in imaging resolution, reaching an imaging resolution of 0.782 μm. According to the measured resolution and the NA, the *k* factor of the in-inline holography has been approached ~0.502 theoretical limit value, successfully pushing the Abbe diffraction limit.

### Pushing the resolution limit of ultra-high NA ptychography

**Fig. 4:**
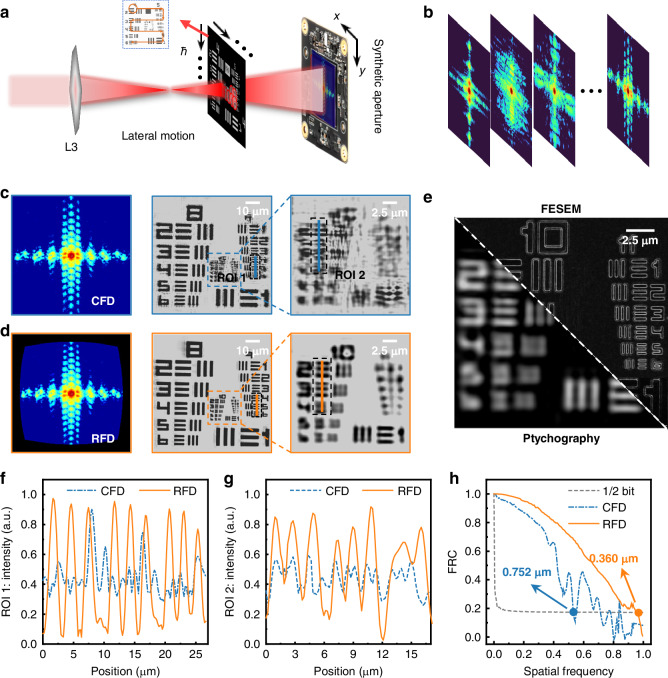
**Experimental results of the ptychographic CDI**. **a** Optical schematic of the ptychography. **b** Experimental raw measured diffraction fields of the brightfield position; **c**, **d** Imaging results by the CFD and the RFD. **e** Imaging results of the ptychography and optical microscope. **f**, **g** The line traces of the different ROIs. **h** the FRC by the CFD and the RFD

Figure 4a, b shows the optical schematic and experimental raw measured diffraction of the brightfield position in the ultra-high NA ptychography, respectively. In addition to the center bright-field diffraction pattern, 8 dark-field diffraction patterns surrounding the bright-field diffraction pattern were captured and stitched together to form a diffraction pattern with an ultra-high NA of 0.88, which represents the highest level in lensless CDIs to date^22,39^. In contrast to the in-line holographic CDI, apart from the diffraction distance error, ptychography is also sensitive to scanning position errors^29^. Therefore, prior to the ptychographic reconstruction, the scanning position errors must be quickly and accurately calibrated (more detailed in Supplementary Note 4). The detailed scanning position error calibration results in Supplementary Fig. S4. Besides the systematic error calibrations, acquiring high-order signals with higher signal-to-noise ratio in the limited detector dynamic range is also challenging to optimize the *k*-factor of the imaging process. Hence, we adopted a high dynamic range (HDR) image fusion technique based on maximum-likelihood estimation to address the problem of insufficient detector dynamic range (more detail in Supplementary Note 5). A series of original Cartesian diffraction signals and HDR diffraction patterns are shown in Supplementary Fig. S5. After model-based computation by the RFD, the momentum-accelerated ptychographic iterative engine (mPIE) was performed 1000 times on the same CPU and GPU also shown in Supplementary Fig. S2. Similar with the experimental results of the holographic reconstruction, the mPIE reconstruction results by the RFD are able to distinguish the linewidth features of the Group 10/Element 3 (resolution: ~0.388 μm) in Fig. 4d, while the results by the CFD without ES effect correction can only resolve the Group 9/Element 4 (resolution: ~0.691 μm) in Fig. 4c. Compared with the truth image measured by field emission scanning electron microscopy (FESEM, Nova NanoSEM 450FP2053/45, FEI), the ptychography imaging results provide good agreement with the FESEM in resolvable linewidth features in Fig. 4e. In the meanwhile, in the FESEM imaging results, the phenomenon of bright and dark linewidths with different contrasts is evident in Group 11. This is due to the poor conductivity of the SiO_2_ substrate, resulting in different electron densities in different linewidth regions in the USAF-1951. Meanwhile, there is also a diagonal misalignment resulting from differences in imaging resolution in Fig. 4e.

Unlike the in-line holographic CDI which focuses on the lower profile in the positive mask, the upper profile of the line trace features must be focused on in the region of interests (ROIs) of Fig. 4f, g because the HIGHRES-1 sample is negative. In the quantitative line traces, the line width feature of Group 9/Element 4 in Fig. 4c is not as distinct as in Fig. 4d. In other words, the resolution of ptychography by the CFD does not reach ~0.691 μm (Group 9/Element 4), while the RFD ptychography can easily resolve ~0.388 μm linewidth features (Group 10/Element 3) in Fig. 4g. Similar to line trace results, the spatial frequency of the FRC^54^ in Fig. 4h shows that the reconstruction result of the ES-effect elimination by the RFD achieves an imaging resolution of 0.360 μm, below the linewidth of the Group 10/Element 3, while the CFD without ES effect correction imaging resolution is only 0.752 μm, beyond the linewidth of the Group 9/Element 4, providing over a twofold resolution enhancement.

Moreover, to test the accuracies of the ES-effect elimination by the proposed model-based computation, we then conducted an experiment comparing RFD with GC approaches. For the curvature distortion corrections of measured diffracted fields from the Cartesian plane to the ES space, the RFD and GC approaches provide consistent mapping geometry. However, for the intensity normalizations of measured diffracted fields, they diverge from each other. In the intensity normalizations by solid angle of the GC approach, it is obvious that this geometric relationship exists only for special scenarios on the coordinate axes (more details in Materials and Methods). For the off-axis solid angle, the geometric relationship will become more complex with an inevitable loss of accuracy. On the contrary, in the intensity normalization via the RFD in the Eq. (11), the rigorous analytical model ensures an accurate intensity normalization relationship. More importantly, even though both approaches can interpolate the measured diffraction pattern from the Cartesian detector to the ES space, the near-field Fresnel diffraction in the GC approach can only solve for the parabolic diffraction distributions, which causes a misalignment between the Fresnel diffraction distributions solved to parabolic space with the measured diffraction pattern interpolated to the ES space. However, the RFD can solve for the diffraction distribution in the ES space for CDIs, and can combine with the measured diffraction pattern interpolated to the ES space to solve the phase retrieval inverse problem directly in the ES space, instead of the conventional Cartesian space. This model-based computation thus once and for all solve the ES effect in high-NA, resolution-limit CDIs. Supplementary Fig. S6 illustrates the results of the RFD and the GC for the ES effect correction in such ultra-high NA setup. It is evident that the GC method for ES effect correction exhibits a discernible decline in measurement resolution, i.e., from Group 10/Element 3 to Group 10/Element 2, in comparison to the RFD for the ES-effect elimination in Fig. S6b. Similarly, as illustrated in Fig. S6c, d, the results of line trace and FRC also demonstrate the superiority of the RFD. Especially in Fig. S6d, the imaging resolution after the ES effect correction via the GC method has degraded to 0.390 μm. At this measured resolution, the imaging factor was calculated as *k* = 0.539, which exceeds the Rayleigh diffraction limit but is considerably higher than the Abbe diffraction limit^47^. However, in the case of the RFD for the ES-effect elimination, the imaging factor is close to the limit of 0.5 (*k* = 0.501), successfully pushing the Abbe diffraction limit. Thanks to the nearly 0.9 ultra-high NA and the Abbe-limit-approaching *k*-factor, the imaging resolution has reached 0.57 times wavelength, which represents the highest level in lensless ptychography to the best of our knowledge.

## Discussion

For the ES effect of the high-NA CDIs, the proposed computational framework of RFD offers a much better solution. Previous results demonstrated that the GC method remains far from the Abbe diffraction-limited *k*-factor even after correcting the ES effect and other imperfect errors^45,47^, which is consistent with our experiment results shown in Supplementary Fig. S6. This is due to the fact that there are space misalignments between diffraction distributions calculated by Fresnel diffraction and interpolated diffraction patterns, and the intensity normalization suffers from more or less loss of accuracy in the geometric corrections in Supplementary Fig. S6c. In contrast, a rigorous propagation model such as RFD yields an explicit mapping of the ES effect and provides a unified space to solve the phase retrieval inverse problem in Supplementary Fig. S6d. By a single inverse spherical projection through rigorous model-based computation before the iteration, the proposed RFD enables to eliminate completely the ES effect in the propagation model of the processing algorithm, including the curvature distortions and intensity normalization. The RFD propagation model is an exact mathematical expression of the RS integral in scalar diffraction theory by just only one FFT solving. It has the exact numerical propagation accuracy of the angular spectrum (usually 2 or 3 FFTs) but does not suffer from transfer function sampling aliasing, and is therefore directly compatible over the entire propagation distance without bandwidth limitations. It also has the computation speed of CFD (also one FFT) but does not suffer from a sudden drop in numerical accuracy in the near Fresnel zone. In the meantime, similar with the CFD, it also breaks the space invariance maintained by the transfer function in angular spectrum propagation, which greatly relaxes the native pixel pitch sampling constraints of the reciprocal space in the observation plane for sub-pixel level imaging resolution in CDIs.

Moreover, irrespective of the in-line holographic CDI or the ptychographic CDI, all experimental results demonstrate that the proposed ES effect elimination by the RFD delivers nearly twice the resolution enhancement. Note that it’s just a coincidence that not all CDI resolutions are improved about 2-fold via the RFD. In fact, the magnitude of resolution enhancement is related to a few complex factors, including specimen characteristics, NA of the CDI, and other errors. From the correction diffraction pattens of Figs. 3d and 4d, it is obvious that the synthetic aperture ptychography has a wider curvature mapping range in the higher-order signals than the in-line holographic CDI, because the NA of the synthetic aperture ptychography is higher than that of the in-line holographic CDI. Similar explanations can be found in the zoom-in imaging results of the in-line holographic CDI and ptychography in Figs. 3d and 4d, i.e., the zoom-in results of in-line holographic CDI are significantly closer to those without ES effect correction relative to the ptychography results, because the high-frequency signals of the diffraction pattern of ptychography are more severely distorted.

In summary, we have demonstrated the theoretical possibility of achieving perfect transfer functions in a variety of NA CDIs, by applying a rigorous Fraunhofer diffraction for the ES effect correction. This novel model-based computational framework perfectly balances varying trade-offs in terms of the accuracy, efficiency, and scalability, which removes the last-mile barrier to achieving the imaging resolution of the Abbe diffraction limit. Similar with other low-NA CDIs^42–44^, we have also achieved the limited imaging factors of the 0.502 in the in-line holographic CDI, successfully pushing the Abbe resolution limit. Then, in the ptychographic prototype, for the first time, we have reached an ultra-high NA of nearly 0.9 by employing the synthetic aperture strategy. In such ultra-high NA setup, we have perfectly eliminated for the ES effect and other errors, optimizing the *k*-factor to 0.501, also pushing the Abbe diffraction limit. Owing to the ultra-high NA and the lowest *k*-factor, we have achieved an imaging resolution of 0.57*λ*. This represents, to the best of our knowledge, a record wavelength-to-resolution ratio for real-world object in lensless CDIs.

Generally speaking, the RFD computational framework belongs to the scalar diffraction theory^55,56^. Therefore, it can be applied to extensive realms involving forward and backward diffraction propagation, optical spatial imaging, phase retrieval of the inverse problems, wavefield communications and sensing, as well as optical calculations and encryption. Naturally, the wave fields are not restricted to electromagnetic fields. Applications in related fields dealing with wave propagation such as acoustics, seismology and wave mechanics can also be expected to be plentiful.

Similarly, the proposed computational framework focuses only on the rigorous modeling of forward-backward diffraction to eliminate the ES effect in CDIs, which makes it perfectly compatible with existing solutions for specific problems. In fact, we have combined the proposed framework with systematic parameter calibration strategies. In the future, the proposed framework will be further combined with existing methods for high-fidelity reconstruction and robust inversion in spatio-temporally partially coherent sources and high-noise difficult datasets.

## Materials and methods

### ES effect correction

Free-space optical field propagation endeavors to address the homogeneous Helmholtz wave equation within certain boundary conditions, either through exact solutions or approximations. In the scalar diffraction theory, the RS integral eliminates the lack of self-consistency of the boundary conditions of the Kirchhoff integral in ultra-near-field diffraction, and its mathematical structure is sufficiently simple that it is often considered to be an exact solution of the Huygens-Fresnel diffraction. In scalar diffraction theory, the mathematical expression for the rigorous RS diffraction integral is given by

4 $$U(x,y,z)=\frac{z}{i\lambda }{\iint }_{\Sigma }U(\xi ,\eta ,0)\frac{\exp (ikr)}{{r}^{2}}d\xi d\eta$$

From the geometric relation in Fig. 2b, the relative distance *r* of the source and observation points can be expressed as

5 $$r=\sqrt{{z}^{2}+{(x-\xi )}^{2}+{(y-\eta )}^{2}}$$

The square root of the distance term makes the solution of the RS integral difficult. Therefore, by a binomial expansion analysis, i.e., the Maclaurin series, the relative distance can be expanded as

6 $$r=z\left[1+\frac{{(x-\xi )}^{2}}{2{z}^{2}}+\frac{{(y-\eta )}^{2}}{2{z}^{2}}-\frac{{(x-\xi )}^{4}}{8{z}^{4}}-\frac{{(y-\eta )}^{4}}{8{z}^{4}}+\cdots \right]$$

When only the first two orders are retained, the radiation wave are already assumed to be parabolic, not spherical in physical. Further, using the paraxial approximation (*r* ≈ *z*) in the denominator, the Fresnel diffraction integral in the GC can be simplified by the RS diffraction integral in the near-filed region as

7 $$\begin{array}{l}U(x,y,z)=\displaystyle\frac{\exp (ikz)}{i\lambda z}{\iint }_{\Sigma }U(\xi ,\eta ,0)\exp \left\{\frac{ik}{2z}[{(x-\xi )}^{2}+{(y-\eta )}^{2}]\right\}d\xi d\eta \\\qquad\qquad\; =\frac{\exp (ikz)}{i\lambda z}\exp \left[\frac{ik({x}^{2}+{y}^{2})}{2z}\right]{\iint }_{\Sigma }U(\xi ,\eta ,0)\exp \left[\frac{ik({\xi }^{2}+{\eta }^{2})}{2z}\right]\exp \left[-\frac{i2\pi }{\lambda z}(x\xi +y\eta )\right]d\xi d\eta \end{array}$$

Commonly, the integration region ∑ in the source plane is extremely small in scalar diffraction region, geometrically much smaller than the propagation distance *z*, which will directly lead to the fact that *ξ*^2^ and *η*^2^ in the quadratic exponential term can be mathematically ignored. At this case, the parabolic wave is approximated as a plane wave, and it breaks the space invariance maintained by the transfer function in angular spectrum propagation and greatly relaxes the native pixel pitch sampling constraints of the reciprocal space in the observation plane for sub-pixel imaging. Therefore, the CFD integral can be approximated as in the Cartesian plane

8 $$U(x,y,z)=\frac{\exp (ikz)}{i\lambda z}\exp \left[\frac{ik({x}^{2}+{y}^{2})}{2z}\right]{\iint }_{\Sigma }U(\xi ,\eta ,0)\exp \left[-\frac{i2\pi }{\lambda z}(x\xi +y\eta )\right]d\xi d\eta$$

However, the mechanism of the paraxial approximation determines that the CFD is only able to keep the accuracy of the RS diffraction integral in the far-field region, and when the detector is in the Fresnel region of the near-field, the CFD integrals are no longer applicable because of the non-negligible optical field curvature distortion between the Cartesian plane detector and the real ES. Therefore, starting from the RS diffraction integral once again, based on the Taylor expansion, the more accurate relative distance *r* can be rewritten as

9 $$\begin{array}{l}\displaystyle r=\sqrt{{z}^{2}+{x}^{2}+{y}^{2}}+\frac{\partial r}{\partial \xi }\xi +\frac{\partial r}{\partial \eta }\eta +\frac{1}{2}\frac{{\partial }^{2}r}{\partial {\xi }^{2}}{\xi }^{2}\\\quad\;\;\;+\,\frac{1}{2}\frac{{\partial }^{2}r}{\partial {\eta }^{2}}{\eta }^{2}+\frac{{\partial }^{2}r}{\partial \xi \partial\eta }\xi \eta +\cdots\end{array}$$

Correspondingly, ∂*r*/∂*ξ* and ∂*r*/∂*η* can be expressed as

10 $$\begin{array}{cc}\displaystyle\frac{\partial r}{\partial \xi }=-\frac{x}{\sqrt{{z}^{2}+{x}^{2}+{y}^{2}}}, &\displaystyle\frac{\partial r}{\partial \eta }=-\frac{y}{\sqrt{{z}^{2}+{x}^{2}+{y}^{2}}}\end{array}$$

By a rigorous analytical solution, the RFD can be expressed as Eq. (1). Compared with the CFD of the Cartesian space, the model-based computation gap of diffraction fields in the curvature distortion and the intensity normalization in the real ES space can be expressed as, respectively

11 $$\begin{array}{c}\begin{array}{cc}{x}_{c}=\frac{x}{\sqrt{1+{(x/z)}^{2}+{(y/z)}^{2}}}, & {y}_{c}=\frac{y}{\sqrt{1+{(x/z)}^{2}+{(y/z)}^{2}}}\end{array};\\ \frac{I^{\prime} ({x}_{c},{y}_{c})}{I(x,y)}=\frac{{|U^{\prime} ({x}_{c},{y}_{c},z)|}^{2}}{{|U(x,y,z)|}^{2}}=\frac{{z}^{2}/{r}^{4}}{1/{z}^{2}}=\frac{{z}^{4}}{{({z}^{2}+{x}^{2}+{y}^{2})}^{2}}\end{array}$$

rather than the intensity normalization by solid angle in Fig. 2a

12 $$d{\Omega }_{norm}(x,y)=\frac{d\Omega ({x}_{c},{y}_{c})}{d\Omega (0,0)}=\frac{{z}^{2}dx(dy\,\cos \theta )}{{r}^{2}d\Omega (0,0)}={\left(\frac{z}{\sqrt{{z}^{2}+{x}^{2}+{y}^{2}}}\right)}^{3}\propto \frac{z}{{(\sqrt{{z}^{2}+{x}^{2}+{y}^{2}})}^{3}}$$

where the normalization reflects only the particular geometry on the coordinate axes. For intensity normalization of off-axis solid angle, the GC becomes more complex with a loss of accuracy^45,46,48,49^.

### In-line holographic CDI experimental setup

In-line holographic CDI (also called Fresnel CDI^57^) is a mixture between holography and CDI in which it is common-path scanning in a conical geometry to achieve higher resolution by using lensless phase retrieval. In the experimental prototype, the 632.8 nm illumination beam was emitted from a HeNe laser (N-STP-912, Newport) and passed through the F1 filter (NE10A-A, Thorlabs) and the P1 pinhole (M-ID-1.0, Newport) for beam shaping and brightness control. After a series of reflections, the beam was further shaped and collimated by a beam expander L1 and L2 (GBE05-B, Thorlabs) to reach the plano-convex lens L3 (LA1036-A, Thorlabs). The collimated beam was then passed through lens L3 of the 6 mm focal length, forming an illuminated probe on the surface of the specimen (USAF-1951, #58-198, Edmund Optics) behind the back focal plane. In the meanwhile, the *x-y-z* translation stage (S-C01K016, M-Z03K002, Physik Instrumente) drove the lens L3 to step linearly along the direction of the optical axis with 1 mm/s. Keeping the specimen fixed during the lens step-scanning process, the back-side illuminated CMOS detector (QHY268M, QHYCCD) of the 3.76 μm native pixel, located 18.9 mm from the specimen received a series of axially scanned in-line holograms (region of interest (ROI): 4096×4096 central pixels). It is important to note that the lateral misalignments during the axial scanning of the divergent beam needs to be well calibrated based on cross-correlation registration^33^ by remaining linear axis before the holograms are captured.

### Ptychographic experimental setup

Ptychography is a scanning CDI that combines a large field-of-view and high resolution in the transmission or reflection geometry by moving the illumination probe to various positions. Compared with the adaptive optics^20,58^, its transfer function is perfect theoretically, with resolution being illumination wavelength limited only. Similarly, note that the ptychography imaging, both in the spatial and even Fourier domain, has never been considered as a super-resolution technique since it cannot break the diffraction limit of the imaging system. To validate the scalability for ES effect elimination in ultra-high NA CDI, we developed a near-field ptychography. The optical path structure for beam shaping and brightness control is identical to the in-line holographic CDI system. In contrast, a plano-convex lens L3 (LA1951-A, Thorlabs) of 25 mm focal length was fixed in the propagation path to form the illumination probe. The specimen was mounted on *x-y-z* linear translation stage (S-C01K016, M-Z03K002, Physik Instrumente), where the *x-y* axis derived the specimen (HIGHRES-1, Newport) for lateral scanning in mesh trajectory and the *z*-axis controlled the size of the illuminated probe on the specimen surface. Subject to mechanical interference with the front intercept, the CMOS detector had to be mounted 13 mm from the specimen and could not be further close. To obtain the higher spanning angle, the imaging detector in the ptychography experiment was replaced by another back-side illuminated CMOS (QHY600Pro SBFL, QHYCCD) with a full resolution of 9600 × 6422 and a pixel size of 3.76 × 3.76 µm. The surrounding optical black area features were rejected, and the center 6144 × 6144 pixels were selected as the ROI. The detector was also fixed on another *x-y* translation stage (M-L01K, Physik Instrumente), and drifted 12 mm from the center position for synthetic aperture ptychography between brightfield and darkfield by using the cross-correlation image registration^33^. For higher computational efficiency, we performed a binning operation on all diffractograms^51^.

## Supplementary information

Supplementary Information for Pushing the resolution limit of coherent diffractive imaging

## Data Availability

The complete data that support the plots within this paper and other finding of this study are available from the corresponding authors upon request.

## Code availability

Codes that are used in this paper are not available publicly due to the follow-up research. They are available from the corresponding author upon reasonable request.
