# Supplementary material for: Pushing the resolution limit of coherent diffractive imaging
Source: Light Sci Appl. 2025 Aug 28;14:298. doi: 10.1038/s41377-025-01963-2 (PMC12394597; doi:10.1038/s41377-025-01963-2)
Supplement: Supplementary file 1 — Supplementary Information for Pushing the resolution limit of coherent diffractive imaging [file 41377_2025_1963_MOESM1_ESM.docx]

Supplementary Information for

Pushing the resolution limit of coherent diffractive imaging

Li Liu^1^, Jinxiang Du^1^, Bailin Zhuang^1^, Ming Gong^2^, Jiamin Liu^1^,

Honggang Gu^1,3,4,*^, and Shiyuan Liu^1,2,3,*^

^1^ School of Mechanical Science and Engineering, Huazhong University of Science and Technology, Wuhan, Hubei 430074, China;

^2^ School of Optical and Electronic Information, Huazhong University of Science and Technology, Wuhan, Hubei 430074, China;

^3^ Optics Valley Laboratory, Wuhan, Hubei 430074, China;

^4^ Guangdong HUST Industrial Technology Research Institute, Guangdong Provincial Key Laboratory of Manufacturing Equipment Digitization, Dongguan, Guangdong 523003, China;

*Corresponding author: [hongganggu@hust.edu.cn](file:///C:\Users\li_li\Desktop\科研\2024\Pushing%20the%20Ewald%20sphere%20effect%20resolution%20limit%20in%20coherent%20diffraction%20imaging\nature%20communication\hongganggu@hust.edu.cn); [shyliu@hust.edu.cn](mailto:shyliu@hust.edu.cn)

## Supplementary Note 1: Phase retrievals in various space for CDIs


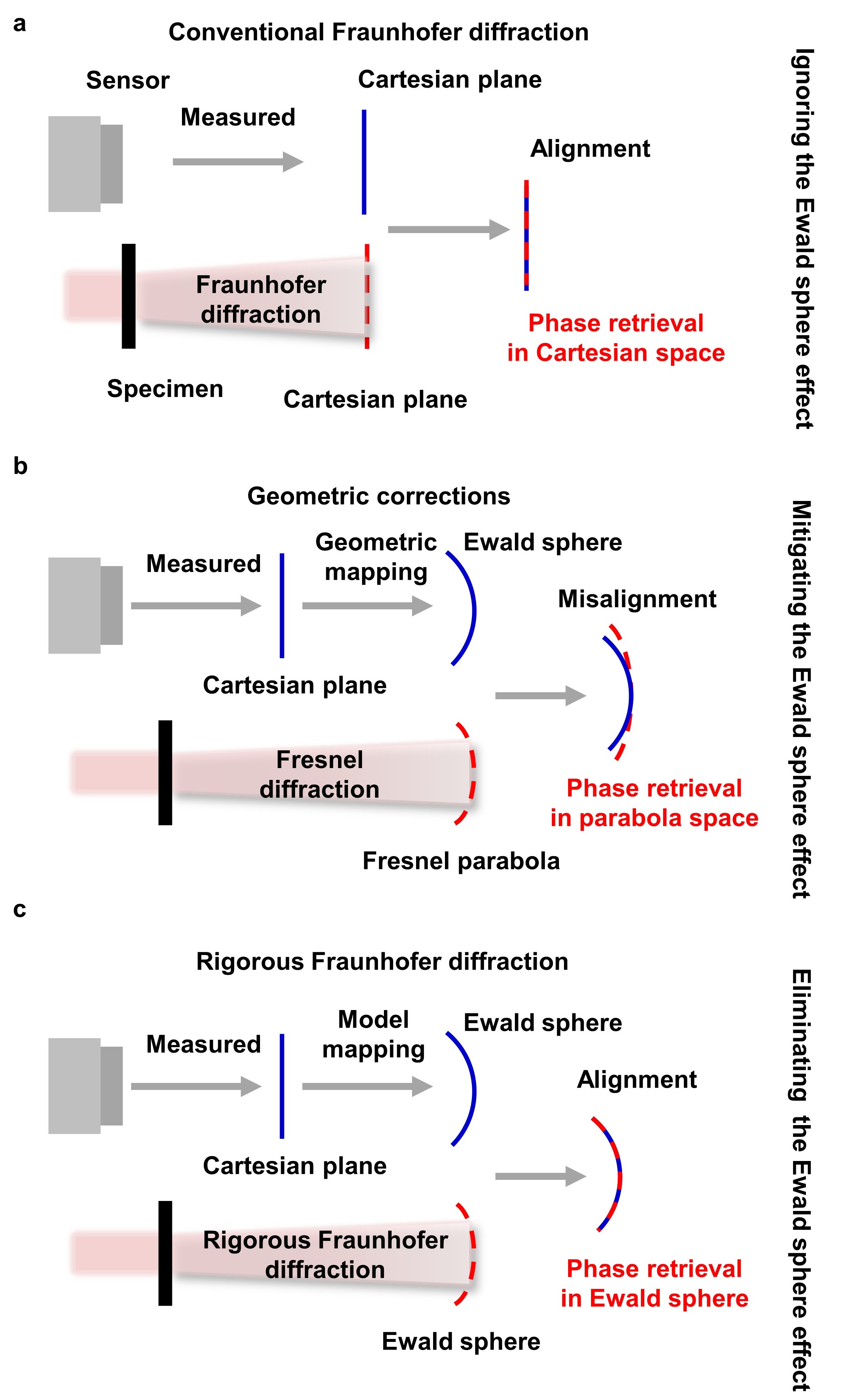


**Fig. S1 Phase retrievals in various space for coherent diffraction imaging (CDI)**. **a,** Cartesian plane. **b,** Fresnel parabola plane. **c,** Ewald sphere plane.

## Supplementary Note 2: Reconstruction algorithm implementations

In this section, we provide detailed instructions on implementing the phase retrieval forward and inverse modeling problems in CDI framework for in-line holographic CDI and ptychography described in the main text.

In the following sections, the notations are defined as follows: the symbol ( ∙ )^T^ , ( ∙ )^∗^, | ∙ |_p_ and ( ∙ )^H^ denote the p-norm, transpose, conjugate and conjugate transpose (Hermitian) operators, respectively. × is the element-wise (Hadamard) multiplication operator, while, | ∙ |, | ∙ |^2^, and / should also be interpreted as element-wise operators. diag( ∙ ) puts the entries of a vector into the diagonal of a matrix. For the convenience of illustration, we follow the convention of linear algebra and vectorize the two-dimensional wavefield as a one-dimensional vector representation. It is important to notice that vectorization is just for simplifying the notation and is not required for computation in practice.

1. **Forward model**

In the forward model, a collimated beam is focused onto the object surface after passing through a plano-convex lens to construct the illumination probe *P*_0_. And the probe *P_n_* diffracts on the surface of a thin object *O* and is propagated by distance *z* to the detector. On account of the relativity of displacement, including the axial or lateral movements of different components, the exit-wave *ψ_n_*(*s*) downstream the object for the *n-*th scanning position can be expressed as:

 (S1)

where, *ћ* stands for the spatial displacement. In the in-line holographic CDI, *ћ* is the axial scanning operation of the lens, but in the ptychography, *ћ* is the lateral scanning operation of the object. Therefore, *ћ* can be written as

 (S2)

AS( ∙ ) stands for angular spectrum propagation operation, and *d_n_* is the axial scanning distance. CS( ∙ ) stands for spatial translation operation, and (*x_n_*, *y_n_*) is the translation coordinate. When the exit-wave *ψ_n_*(*s*) is propagated to the sensor plane, the measurement intensity *I_n_* with respect to the *n-*th scanning position can be expressed as

$I_{n}(u)=\text{ }\left|\left|F\psi_{n}\left(s\right)\right|\right|^{2}+b$ (S3)

where, *s* and *u* are spatial coordinates in the real domain and frequency domain, respectively. *b* is a positive offset matrix of the detector noises. Based on the Ewald sphere (ES) effect elimination, the true diffraction pattern is rewritten as by the rigorous model-based computation gaps

 (S4)

where, *u*_c_ is the real Ewald sphere coordinate, *A* is the factors of the intensity normalization in the Eq. (11), is interpolation operation of the inverse spherical projection and it is a bilinear or bicubic interpolation chosen in proposed computational framework. This is the Step 1 in Fig. S2.


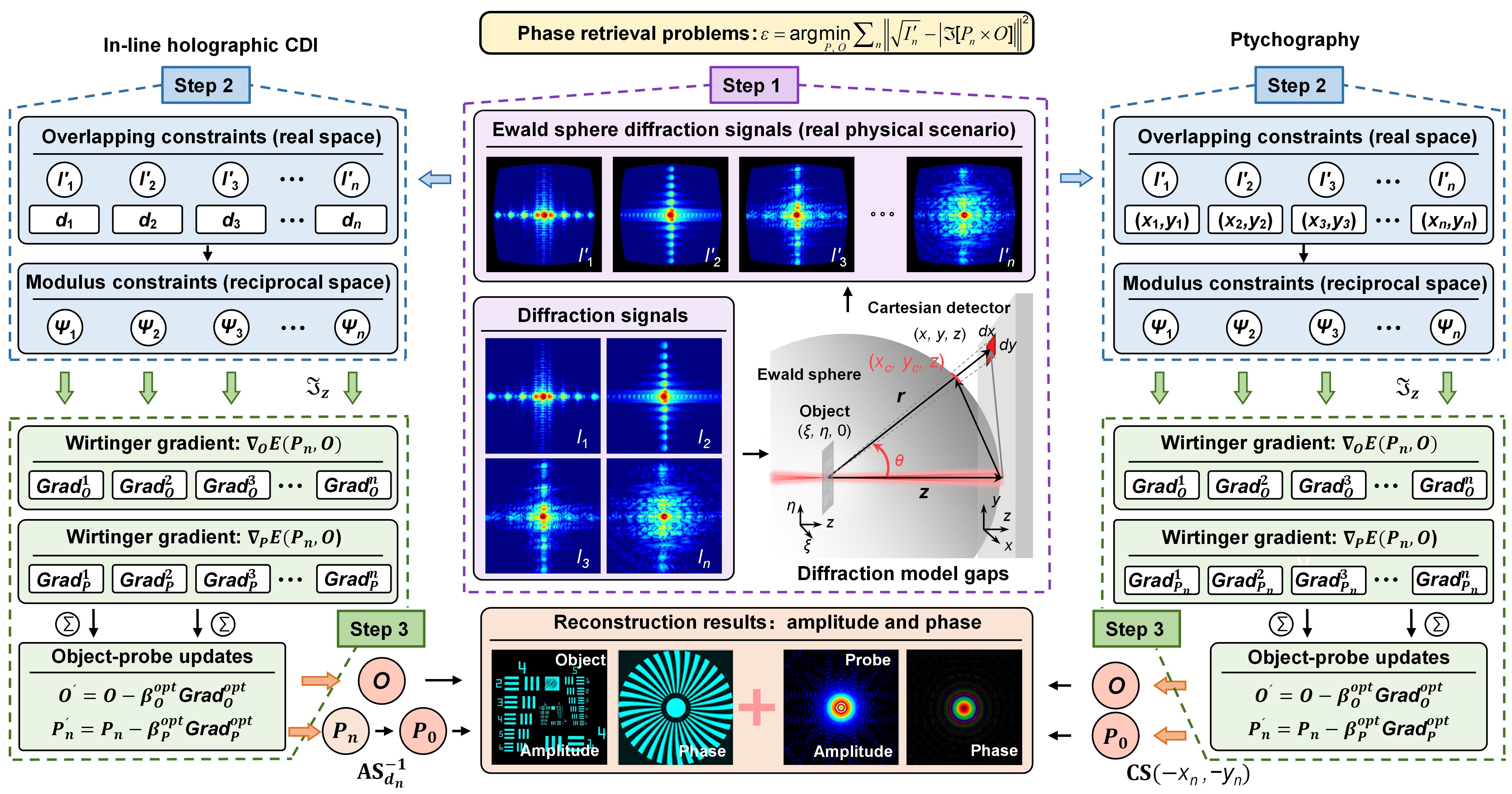


**Fig. S2 The reconstruction algorithms of the in-line holographic CDI and ptychography**

1. **Inverse reconstruction framework for in-line holographic CDI and ptychography**

Based on the phase retrieval inverse modeling problems of the CDIs, we formulate the in-line holographic CDI and ptychography reconstruction as a constrained optimization problem. Generally, the reconstruction framework can be divided into two subtasks, as is shown in Fig. S2.

**Optimization in the reciprocal space.** Search for the optimal wave field intensity *Ψ_n_*(*u*_r_) that minimizes the distance between the diffraction pattern after the ES effect correction *I′_n_* (*u*_c_) and the wave field intensity *Ψ_n_* that satisfies the diffraction model with respect to the near-field exit wave in the forward model of the Eq. (S3).

 (S5)

We choose the amplitude-based fidelity term as the distance metric because of its mathematical similarity to the Poisson noise model [1], which is proven to be an accurate model for noise statistics description. To optimize the fidelity term, we employ the gradient descent method, actively seeking to determine the optimal step size. The Wirtinger gradient is given by

 (S6)

Once the locally optimal update direction is derived, we can perform the gradient descent operation:

 (S7)

where, *β_n_* is the step size for the descent direction. Notice that for specific update direction, the fidelity term can be seen as a convex function with respect to *β_n_*, hence the optimal step size can be calculated by finding the extreme of *F*(*β_n_*):

$\partial F\left(\beta\right)_{n}/\partial\beta_{n}=0$ (S8)

Solving Eq. (S8) yields *β_n_* = 2, thus we derive the optimal wave field for *n-*th scanning position to be

 (S9)

which is equivalent to the well-known modulus constraint projection method. While all *N* scanning positions are traversed, the overlapping constraint projection are also alternating. This is the Step 2 in Fig. S2.

**Optimization in the real space.** Apply a proper update function to the recovered exit-wave *ψ_n_*(*s*) on the surface of the object. The uniqueness about CDIs is that the complex transmission matrix of the probe is not known accurately, so a proper update needs to be conducted on the back-propagated wave field on the surface of the object in order to recover the probe and object wavefield. An error metric is introduced to evaluate the update process

$E=\left\|\psi_{n}^{\text{opt}}\left(s\right)-P_{n}\times O\right\|^{2}$ (S10)

here,

 (S11)

Again, we calculate the Wirtinger gradient with respect to *P_n_* and *O* and take the gradient descent method to optimize *E*:

$\begin{matrix} \nabla E_{P}\left(P_{n}\right)=P_{n}^{*}\times\left(P_{n}\times O-\psi_{n}\left(s\right)\right), \\ \nabla E_{O}\left(O\right)=O^{*}\times\left(P_{n}\times O-\psi_{n}\left(s\right)\right) \end{matrix}$ (S12)

$\begin{matrix} P'_{n}=P_{n}+\beta_{P}\Delta P_{n}=P_{n}-\beta_{P}\nabla E_{P}\left(P_{n}\right), \\ O'=O+\beta_{O}\Delta O=O-\beta_{O}\nabla E_{O}\left(O\right) \end{matrix}$ (S13)

When the *β_P_* = *α_P_*/|*O*|2 max and the *β_O_* = *α_P_*/|*P_n_*|2 max, it is the classic ePIE update function for object and probe update. In order to avoid numerical instability, we adopted to a least-square (LSQ) method [2] to search for optimal step sizes and the resulting LSQ matrix can be expressed as

 (S14)

Where, ℜ denotes the real part of a complex number, and *τ* is a small regularization constant. ℝ is the real number constraint of the step size. Solving the system in Eq. (S14) is computationally cheap, however the cost quadratically grows for a larger number update directions mainly due to the increased number of non-diagonal terms. The computational cost can be significantly reduced if the update directions can be considered mutually orthogonal. In that case, the non-diagonal terms of the matrix in Eq. (S14) become zero and the optimal step sizes can be estimated as

 (S15)

Imposing a constant to avoid the singularity of possible zeros in the denominator, it is a L_2_ regularization [3] on *P_n_* and *O*, which has the equivalent effect as disappearing Tikhanov regularization in the rPIE [4]. After the optimal step sizes are determined, the *P_n_* and *O* can be reconstructed in the Eq. (S13). This is the Step 3 in Fig. S2. Finally, the illumination probe *P*_0_ is reconstructed based on overlapping constraints as

$P_{0}=\left\{\begin{matrix} AS^{-1}(P_{n}^{\text{opt}},\text{ }d_{n}), & \text{in-line holography} \\ CS(P_{n}^{\text{opt}},\text{ }(-x_{n},-y_{n})), & \text{ptychography} \end{matrix}\right.$ (S16)

1. **Derivation of Wringer gradient**

The amplitude-based fidelity term is employed as the loss function *L*, which can be expressed as

$f(u)=L=\frac{1}{2}\left\|\sqrt{I}-Au\right\|_{2}^{2}$ (S17)

where ***A*** is the forward model and *I* is the intensity-only measurement. According to the chain rule of the complex-valued derivations

$\frac{\partial f(u)}{\partial u}=\frac{\partial f(u)}{\partial L}\frac{\partial L}{\partial u}=L^{T}\frac{\partial L}{\partial u}=L^{T}\left(\frac{\partial L}{\partial\left(Au\right)}\frac{\partial\left(Au\right)}{\partial u}+\frac{\partial L}{\partial\left(Au\right)^{*}}\frac{\partial\left(Au\right)^{*}}{\partial u}\right)$ (S18)

here,

$\begin{matrix} \frac{\partial L}{\partial\left(Au\right)}=\frac{\partial\sqrt{\left|Au\right|^{2}}}{\partial\left(\left|Au\right|^{2}\right)}\frac{\partial\left(\left(Au\right)^{*}\times Au\right)}{\partial\left(Au\right)}=\frac{1}{2}diag\left(\frac{1}{\sqrt{\left|Au\right|^{2}}}\right)diag\left(Au\right)^{*}, & \frac{\partial\left(Au\right)^{*}}{\partial u}=0 \end{matrix}$ (S19)

Combining the above formulas, we can obtain the derivative *f* with respect to ***u***

$\frac{\partial f\left(u\right)}{\partial u}=\frac{1}{2}L^{\text{T}}diag\left(\frac{1}{\sqrt{\left|Au\right|^{2}}}\right)diag\left(Au\right)^{*}A$ (S20)

the gradient is thus given by [5]

$\nabla f\left(u\right)=\left(\frac{\partial f\left(u\right)}{\partial u}\right)^{*}=\frac{1}{2}A^{*}diag\left(Au\right)diag\left(\frac{1}{\sqrt{\left|Au\right|^{2}}}\right)L$ (S21)

## Supplementary Note 3: Autofocusing strategy for diffraction distance error

1. **Algorithm implementation**

If the diffraction distance error occurs in the iterative process, the *i-*th iteration of the updated object *O_i_* will reconstruction artefacts because of the uncertainty in scaling of the reconstruction pixel size after the Eq. (S13). Therefore, to obtain accurate diffraction distance between the object and detector in the CDIs, the estimated object is propagated to *k* distances around the reconstruction plane using an angular spectrum propagator given by

$O_{i}\left(k\right)=AS\left(O_{i},\text{ }k\Delta z\right)$ (S22)

Note that the propagation interval Δ*z* is chosen to be on the order of the depth of field (DOF). Here, the DOF can be expressed as

$\Delta z=\lambda/\left(2z/D\right)^{2}$ (S23)

where, *D* is the physical size of detectors and *z* is the diffraction distance. Hence, the sharpness of the propagated object is evaluated using the total variation (TV) functional as

$S\left(k\right)=\iint\sqrt{\left|\partial_{x}O_{i}\left(k\right)\right|^{p}+\left|\partial_{y}O_{i}\left(k\right)\right|^{p}+\sigma}$ (S24)

where, *σ* is a very small value set to prevent the results of matrix operations from falling into singular values. When *p* = 2, it will be an isotropic diffusion model, i.e., zPIE sharpness metric. When 1< p < 2, The variogram from Eq. (S24) is chosen to design an adaptive operator that can adjust the p-value according to the intensity distribution of each pixel, i.e., adaTV-PIE sharpness metric. The designed Lp-norm based model is adaptive with each image pixel:

$p\left(x,y\right)=1+\frac{1}{1+\left(\frac{\nabla\left(F^{-1}\left(W\left(u,v\right)\right)\right)}{dx}\right)^{2}+\left(\frac{\nabla\left(F^{-1}\left(W\left(u,v\right)\right)\right)}{dy}\right)^{2}}$ (S25)

where, ∇ and *F*^-1^ represent the gradient operator and the inverse Fourier transform operator, respectively. *W* (*u*, *v*) is the expression of the Wiener filter method in the frequency domain. The form of Wiener filter is referred to acquire the inversion data around the set diffraction distance, which is used as the parameter of adaptive Lp-norm:

$W\left(u,v\right)=\frac{H_{z}^{*}\left(f_{x},f_{y},\Delta z\right)}{\left|H_{z}\right|^{2}+P_{N}\left(u,v\right)/P_{S}\left(u,v\right)}\times G\left(u,v\right)$ (S26)

where, *H_z_* denotes the point spread function (PSF), * denotes conjugate, *G* denotes the degraded image in frequency domain, *P_N_*(*u*,*v*) / *P_S_*(*u*,*v*) is the noise to signal power ratio. Based on the sharpness metric in the Eq. (S24), the second-order Taylor series in *z* can be expressed as

$S\left(z\right)=S\left(z'\right)+\left(z-z'\right)\partial_{z}S\left(z'\right)+\frac{1}{2}\left(z-z'\right)^{2}\partial_{z}^{2}S\left(z'\right)+\cdots$ (S27)

where *z'* is an arbitrary point. At the optimum *z*^opt^, the first-order term vanishes:

$S\left(z\right)\approx S\left(z^{\text{opt}}\right)++\frac{1}{2}\left(z-z^{\text{opt}}\right)^{2}\partial_{z}^{2}S\left(z^{\text{opt}}\right)$ (S28)

It is the typical symmetric form around *z*^opt^ to second order. The feedback term should be zero at the optimum, and it can be expressed as

$\delta z=\eta\delta z+c\left(\sum_{k} S\left(k\right)k\Delta z\right)/\sum_{k} S\left(k\right)$ (S29)

In addition to the feedback term, the search direction *δz* has a damped momentum term *η*, which allows the algorithm to accelerate the search in the case of repeated steps in the same direction. We note that the search can be accelerated by increasing the proportionality factor *c* in front of the feedback term. According to the search direction, the object-detector distance *z* in the (*i+1*)*-*th iteration can be expressed as

$z_{i\text{+1}}=z_{i}-\delta z$ (S30)

Performing Eqs. (S24) - (S30) until the object-detector distance diffraction converge and are no longer oscillate, the autofocusing algorithm will output the diffraction distance *z*, the amplitude and phase reconstruction results of the object and the probe.

1. **Calibration results**

We performed 500 iterations of the zPIE [6] and the adaTV-PIE [7] algorithms in the in-line holographic CDI, respectively. Compared with the direct reconstruction results without the diffraction distance error calibration in Fig. S3a, the results of the zPIE and adaTV-PIE have the marked improvement in Figs. S3b and S3c. Further comparing the reconstruction results of the zPIE and adaTV-PIE in Figs. S3b and S3c, we find that the reconstruction artefacts are almost eliminated in the adaTV-PIE, while the reconstruction results of the zPIE still have some misalignment artefacts in high resolution line-pairs. Fig. S3d shows the line traces of the zPIE and adaTV-PIE reconstruction results. The adaTV-PIE reconstruction does have a higher resolution because it is adaptive and is able to adjust to each pixel of the reconstructed image at each iteration, thereby better preserving the details of the specimen. Also, with different initial diffraction distances to start the iteration, the adaTV-PIE has faster convergence speed and convergence accuracy in Fig. S3e. This strategy has also been adopted in ptychography for diffraction distance error calibration, which will not be repeated here.

**
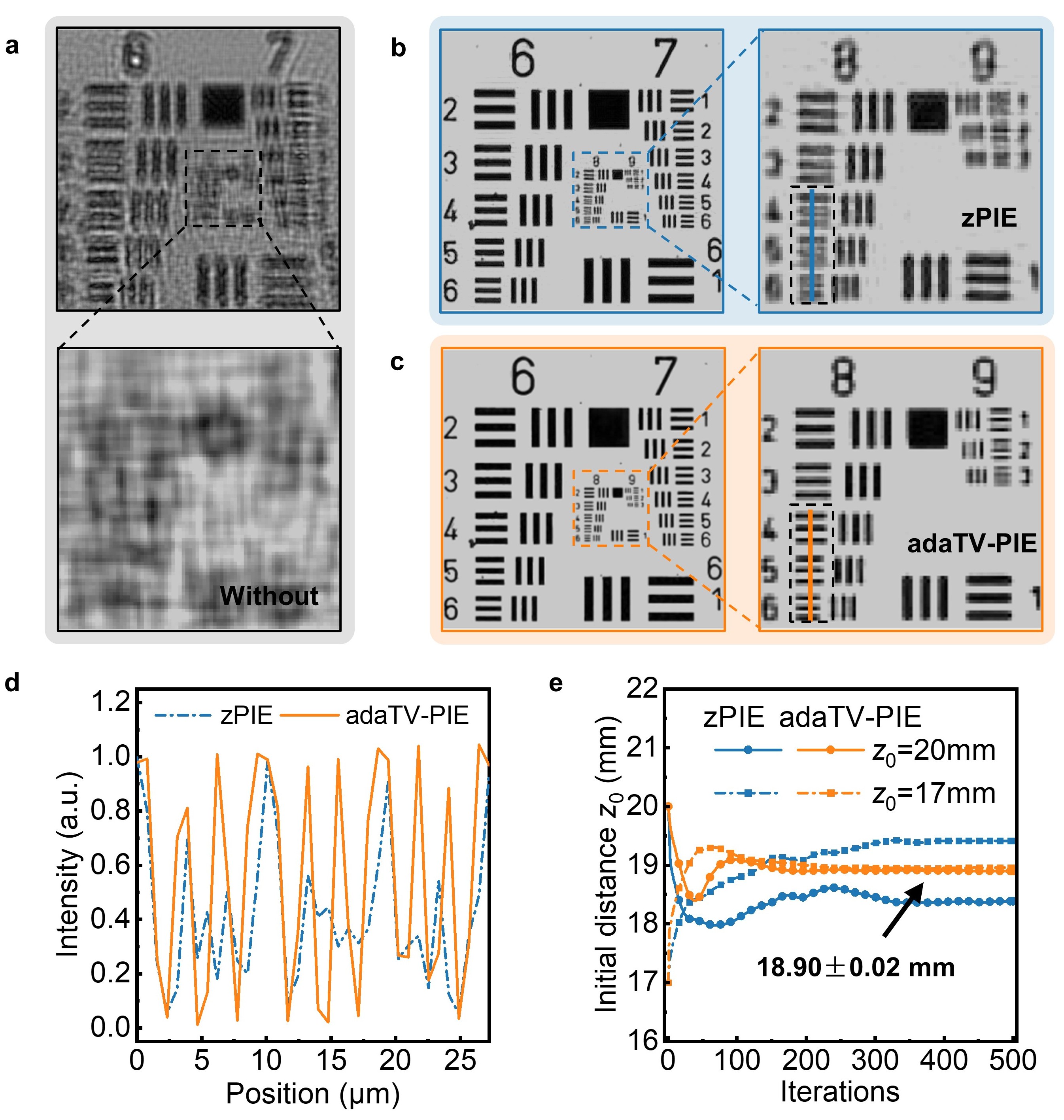
**

**Fig. S3 Experimental results of the diffraction distance error calibration in the in-line holographic CDI**. **a,** Without calibration. **b,** zPIE autofocusing strategy. **c,** adaTV-PIE autofocusing strategy. **d,** Line traces. **e,** Convergence results versus different initial distances *z*_0_.

## Supplementary Note 4: Parallel cross-correlation strategy for translation position errors

1. **Algorithm implementation**

If the position errors occur in the scanning shift process, the (*i*+1)-th iteration of the updated object *O j i+1* will move to the correct measurement positions relative to the *i-*th iteration of the object *O j i*, resulting in reconstruction artefacts in the iteration process. Therefore, the centrosymmetric and axisymmetric virtual probe array is constructed around origin shift *Rj i*(0) after the Eq. (S13) in the *i-*th iteration as

$\begin{matrix} P_{i}^{j}(s-R_{i}^{j}(k))=P_{i}^{j}(s-R_{i}^{j}(0))+ S_{k}, & k=0,\text{ }1,\text{ }2,\text{ }\cdots\end{matrix}$ (S31)

where, *Rj i*(0) is the *j-*th scanning shift of the called center probe. *S_k_* are the 1-pixel offset in each direction relative to the origin shift and *k* is the offset number. *Rj i*(*k*) are virtual array probe offsets derived from the origin offset of the center probe, including but not limited to the square array in the mesh trajectory or circular arrays in the Fermat trajectory.

Since the object cross-correlation information before and after updating can provide a feedback signal for the correct position [8], the cross-correlations between the reconstructed object *O j i+1* in the each array probe *Pj i*(*s* - *Rj i*(*k*)) of the (*i+1*)-th iteration and the reconstructed object *O j i* in the center probe *Pj i*(*s* - *Rj i*(0)) of the *i-*th iteration are recorded respectively as

 (S32)

where, Π(*s* - *Rj i*(*k*)) denotes the binary function within the regions of the object illuminated by each probes. After the normalization operation, the parallel cross-correlation peaks are further weighted by the activation function to accelerate its iterative direction towards correct motion positions as

$\begin{matrix} pCC(k)=sigmoid\text{(}norm\text{(}CC\text{(}k\text{))),} & sigmoid(x) \end{matrix}=\frac{1}{1+e^{-x}}$ (S33)

where, the norm function is the normalization function and *pCC*(*k*) are the weighted cross-correlation peaks of each array probe. The sigmoid weighting strategy is not the only option, and alternative activation functions with gradient differences, such as the hyperbolic tangent (*tanh*) weighting, can also produce equivalent effects.

According to the weighted parallel cross-correlation peaks, the relative shift *ej i* of the array probes can be obtained as

$\begin{matrix} e_{i}^{j}=\beta_{1}\times\sum_{k} \text{[}pCC\text{(}k\text{)}\times S_{k}], & k=\text{0, 1, 2, }\cdots\end{matrix}$ (S34)

where, *β*_1_ is the feedback parameter. In general, the recommended value is more than 50. Therefore, the updated center probe shift, i.e., the scanning position of the planar translation stage, in the (*i*+1)-th iteration can be expressed as

$R_{i+1}^{j}(0)=\beta_{2}R_{i}^{j}(0)+(1-\beta_{2})e_{i}^{j}$ (S35)

where, *β*_2_ is the exponential decay rate between 0 and 1. Due to increase the inertia maintenance and environmental sensing of the Adam optimizer or the momentum thought, the sensitivity of the parameters *β*_1_ and *β*_2_ to the convergence result will decrease dramatically. By performing Eqs. (S31) - (S35) until all scan positions converge and are no longer oscillate, the ptychographic self-calibration algorithm will output the translation position errors ∆*R*(∆*x*, ∆*y*) of the planar translation stage, the amplitude and phase reconstruction images of the object and the probe.

1. **Calibration results**

We performed 500 iterations of the translation position error calibration algorithms [9] in the ultra-high NA ptychography. It can be clearly seen that the conventional mPIE algorithm does not deal with the scanning position errors in the translation stage, which leads to a significant degradation of the imaging resolution in Fig. S4a. However, a compelling contrast is observed when comparing the mPIE algorithm with the position error self-calibration algorithms, which effectively discriminate the 0.388 µm line width features of Group 10/Element 3 in Fig. S4b. Detailed visualizations of the translation position map, depicted in Fig. S4c, provide insights between the initial positions and the calibrated convergence positions obtained from the parallel cross-correlation algorithm. In the map, the initial positions are represented by blue balls, while the orange balls indicate the calibrated convergence positions. Based on the translation position map, the translational position errors in the X and Y directions can be calculated as shown in Figs. S4d and S4e, respectively, which clearly shows that the imaging resolution of the ptychography is unable to break through the positioning error of the linear translation stage.

**
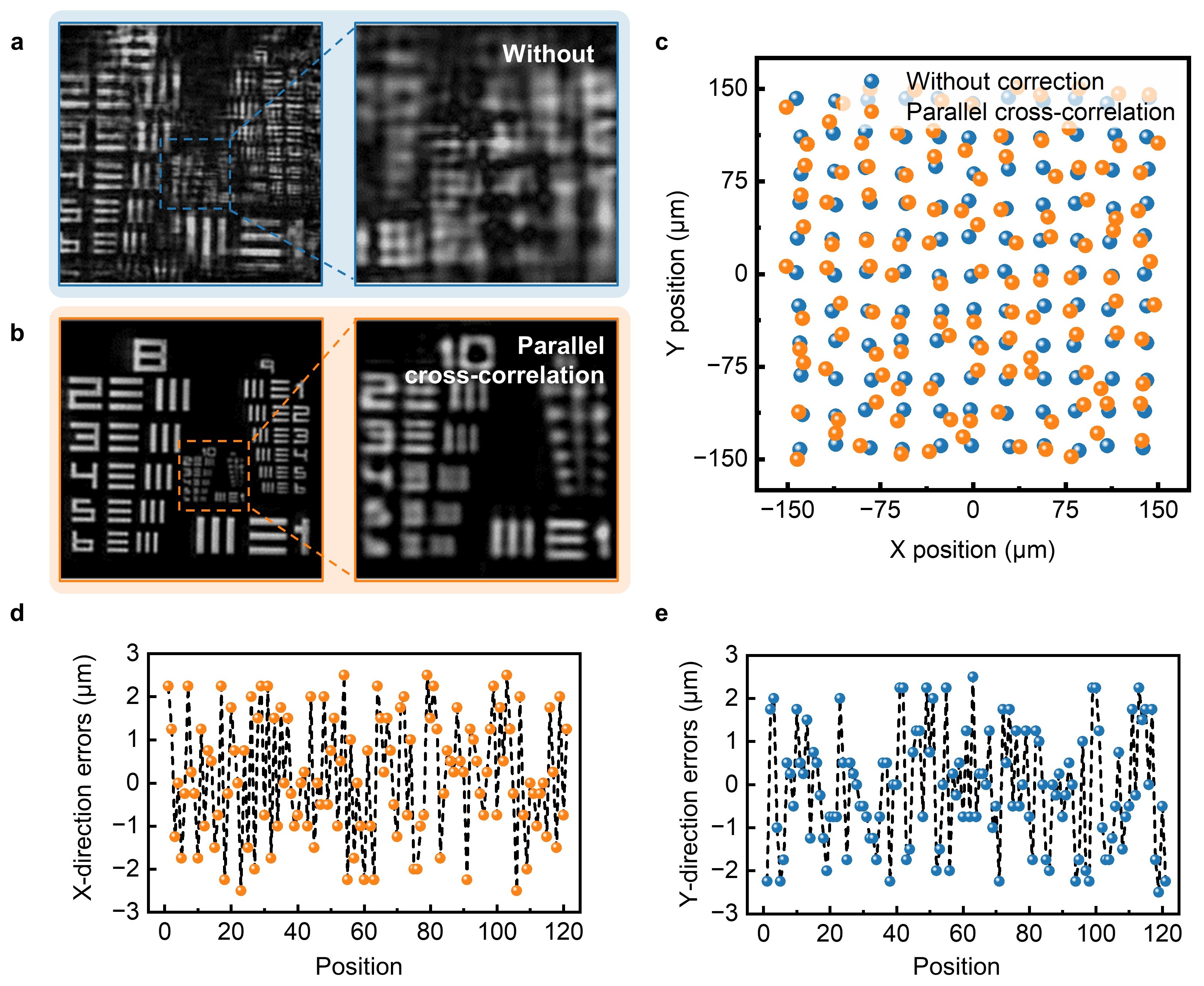
**

**Fig. S4 Experimental results of the translation position error calibration in the ptychography**. **a,** Without calibration. **b,** Parallel cross-correction strategy. **c,** Calibration results of the position error. **d,** X-direction position errors. **e,** Y-direction position errors.

## Supplementary Note 5: Maximum-likelihood high-dynamic-range (ML-HDR) diffractogram fusion

1. **Algorithm implementation**

Whether it is a complementary metal-oxide-semiconductor (CMOS), charge-coupled device (CCD) or other detectors, the optical intensities are inevitably contaminated by noises during the acquisition process. Therefore, the establishment of a complete detector noise model is of great significance for the error tracing and the signal processing. During the exposure time *t_i_* (*i* = 1, 2, …, *N*), the relationship between the digital readout *Z_i_* and the optical irradiance of the detector can be expressed as

$Z_{i}=\left\{\begin{matrix} 0 & 0\leq E<E_{\text{threshold-min}} \\ g(Et_{i}+Dt_{i}+R) & E_{\text{threshold-min}}\leq E<E_{\text{threshold-max}} \\ Z_{\max} & E_{\text{threshold-max}}\leq E \end{matrix}\right.,$ (S36)

where, *E* is the optical irradiance coupled with the photon shot noise (PSN), *D* is the dark current shot noise (DCSN) from photons to charges, *R* is the reading noise (RN) from charges to the digital readout, and *g* is the overall gain coefficient during the conversion process. Due to the inherent properties of the quantum efficiency and full well effect of detectors, the digital readout can be recorded in a finite dynamic range between the minimum and maximum optical irradiances [*E*_threshold-min_, *E*_threshold-max_]. Under the influence of Poisson noises (i.e., the PSN and DCSN) and Gaussian noises (i.e., the RN), mathematical expectation *μ_Zi_* and variance σ^2^ *Zi* of the digital readout can be calculated respectively, as given by

$\begin{matrix} \mu_{Z_{i}}=g(\mu_{E}t_{i}+\mu_{D}t_{i}+\mu_{R}), \\ \sigma_{Z_{i}}^{2}=gt_{i}(\mu_{E}+\mu_{D})+g^{2}\sigma_{R}^{2}, \end{matrix}$ (S37)

where, *μ_E_*, *μ_D_* and *μ_R_* are the mathematical expectations for the PSN, DCSN and RN respectively, and σ*2 R* is the variance of the RN. Similarly, the digital readout *B_i_*, the mathematical expectation *μ_Bi_* and the variance σ*2 Bi* of the dark frames during the exposure time *t_i_* without the optical irradiance can be calculated respectively by

$\begin{matrix} B_{i}=g(Dt_{i}+R), \\ \mu_{B_{i}}=g(\mu_{D}t_{i}+\mu_{R}), \\ \sigma_{B_{i}}^{2}=gt_{i}\mu_{D}+g^{2}\sigma_{R}^{2}. \end{matrix}$ (S38)

A joint distribution between the diffraction frames, the dark frames, and the optical irradiance *E* can be established as given by

$\begin{matrix} \mu_{Y_{i}}=0, \\ \sigma_{Y_{i}}^{2}=2g(\mu_{E}t_{i}+\mu_{D}t_{i})+2g^{2}\sigma_{R}^{2}. \end{matrix}$ (S39)

where, (*E_Zi_* - *E*) and (*D_Zi_* - *D_Bi_*) obey the Skellam distribution, and (*R_Zi_* - *R_Bi_*) obeys the Gaussian distribution. When the optical irradiance, namely Poisson eigenvalue, is very large, the Skellam distribution can be approximated as the Gaussian distribution. Therefore, in this case, the mathematical expectation and variance of the joint distribution can be calculated as

$\text{P}\left(\left.y_{i}\right|\mu_{Y},\sigma_{Y_{1}}^{2},\sigma_{Y_{2}}^{2},\cdots,\sigma_{Y_{i}}^{2}\right)=\prod_{i=1}^{N} \text{P}\left(\left.y_{i}\right|\mu_{Y},\sigma_{Y_{i}}^{2}\right),$ (S40)

In the above mathematical derivation of the joint distribution, the conditional probability is equal to the joint probability of the joint distribution, as given by

$R_{i+1}^{j}(0)=\beta_{2}R_{i}^{j}(0)+(1-\beta_{2})e_{i}^{j}.$ (S41)

and the maximum likelihood function of the absolute irradiance ${\hat{\text{μ}}}_{\text{E}}$ can be expressed as

$\hat{\mu}_{E}\text{= arg }\mathop{\text{max}}\limits_{\mu_{E}} \prod_{i=1}^{N} \text{P}\left(\left.y_{i}\right|\mu_{Y},\sigma_{Y_{i}}^{2}\right)\propto\text{arg }\mathop{\text{max}}\limits_{\mu_{E}} \left(\text{exp}\left(\sum_{i=1}^{N} \frac{\left(Z_{i}-B_{i}-gt_{i}E\right)^{2}}{-2\sigma_{Y_{i}}^{2}}\right)\right).$ (S42)

Where, *N* is the number of exposures. According to the first-order partial derivative of log-likelihood functions (∂*P*/∂*E*=0), the maximum likelihood estimation of the absolute irradiance can be derived as

$\hat{\mu}_{E}\text{=}\frac{\sum_{i=1}^{N} \left(t_{i}^{2}/\sigma_{Y_{i}}^{2}\right)\left(Z_{i}-B_{i}\right)/\left(gt_{i}\right)}{\sum_{i=1}^{N} \left(t_{i}^{2}/\sigma_{Y_{i}}^{2}\right)}=\frac{\sum_{i=1}^{N} w_{i}\left(Z_{i}-B_{i}\right)/\left(gt_{i}\right)}{\sum_{i=1}^{N} w_{i}}.$ (S43)

It’s obvious that the Eq. (S43) is a weighted-normalization expression, where the weighting factor *w_i_* is

$w_{i}\text{=}\frac{t_{i}^{2}}{\sigma_{Y_{i}}^{2}}=\frac{t_{i}^{2}}{gt_{i}\mu_{E}+\sigma_{B_{i}}^{2}}.$ (S44)

On the other hand, from the diffraction frames and dark frames of the Eq. (S36) and Eq. (S38), we can approximately estimate the mathematical expectations of the absolute irradiance, as given by

$\mu_{E}=\sum_{i=1}^{N} \frac{Z_{i}-B_{i}}{Ngt_{i}}.$ (S45)

When further taking the response characteristics of the detector at different exposure times into account of the Eqs. (S38) and (S45), and the weighting factor *w_i_* of the Eq. (S44) can be optimized as

$w_{i}=t_{i}^{2}/\left(t_{i}\sum_{i=1}^{N} \frac{Z_{i}-B_{i}}{Nt_{i}}+\sigma_{B_{i}}^{2}\right),$ (S46)

Through further in-depth analysis of the weighting factor *w_i_* in the Eq. (S46), we find that the weighting function suppresses the signal proportion under low exposure time. Therefore, for the case of low exposure time, although the Skellam distribution cannot be approximated simply as the Gaussian distribution, the optimized weighting function remains the physical applicability.

1. **Experiment results**


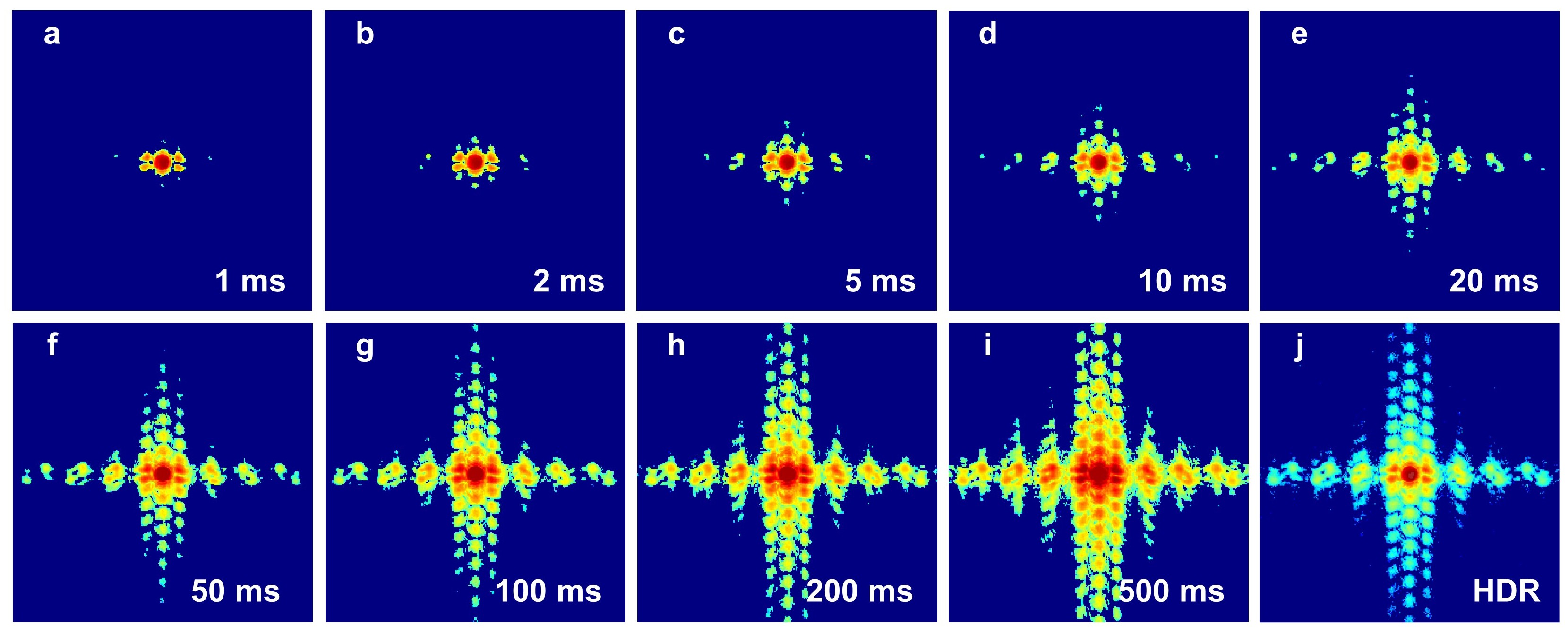


**Fig. S5 Measured diffraction frames versus different exposure times. a,** 1 ms. **b,** 2 ms. **c,** 5 ms. **d,** 10 ms. **e,** 20 ms. **f,** 50 ms. **g,** 100 ms. **h,** 200 ms. **i,** 500 ms. **j,** ML-HDR absolute irradiance.

We performed the ML-HDR diffractogram fusion technology [10] for the all-diffraction signals with 1 ms, 2 ms, 5 ms, 10 ms, 20 ms, 50 ms, 100 ms, 200 ms, and 500 ms. The diffraction frames of the one of scanning position are collected by the CMOS detector and are shown in Figs. S5a- S5i. Meantime, the dark frames were also collected with same exposure times. Based on the direct measurement parameters of the diffraction frames, dark frames and exposure times, the fusional absolute irradiance by the ML-HDR method is shown in Fig. S5j. From Figs. S5a- S5i, it can be clearly seen that as the exposure time increases, the diffraction information in the high Q regions gradually exposes, in the meanwhile, the low-frequency diffraction information is severely overexposed. The collected diffraction fields can only reflect its truth in a limited range. When the ML-HDR image fusion technology is applied to ptychography imaging, Fig. S5j shows more details in the ptychography compared with single exposures. Likewise, this technology has also been applied in in-line holographic CDI to extend the effective NA to enhance the imaging resolution.

## Supplementary Note 6: Comparison experiments of the geometric corrections and the proposed rigorous Fraunhofer diffraction for the Ewald sphere effect

In fact, all scattering vectors end on the Ewald sphere owing to energy conservation. Thus, the diffraction pattern also lies on a sphere centered at the object. Note that it is not sufficient to rescale the intensity normalization based on the fact that the intensity falls by 1/*r*^2^ [11,12], because besides the intensity scaling, the geometric area of the interpolated region is also changes. Therefore, for a pixel (e.g., the red square in Fig. 2a with the red coordinate set) characterized by its lateral size d*x* and d*y* at a position (*x*, *y*) on a planar detector at *z* the solid angle *d*Ω (*x*_c_, *y*_c_) is expressed in Cartesian coordinate

$d\Omega\left(x_{c},y_{c}\right)=\frac{z^{2}dx(dy\cos\theta)}{r^{2}}=\left(\frac{z}{\sqrt{z^{2}+x^{2}+y^{2}}}\right)^{3}\propto\frac{z}{\left(\sqrt{z^{2}+x^{2}+y^{2}}\right)^{3}}.$ (S47)

Using this formula, a matrix of solid angles *d*Ω (*x*_c_, *y*_c_) are computed for all detector pixels. Obviously, the solid angle per pixel decreases for pixels being further away from the center and would thus measure a lower intensity compared to the equally illuminated surface elements on the corresponding sphere. For the experimental data the resulting matrix of solid angles is normalized [13,14]

$d\Omega_{norm}\left(x,y\right)\to\frac{d\Omega\left(x_{c},y_{c}\right)}{d\Omega\left(0,0\right)}\text{，}$ (S48)

where dΩ(0, 0) denotes the solid angle of the central pixel. However, the matrix of solid angles just reflects the particular scenarios on the coordinate axes of the change in lateral size *dy*. In the off-axis scenarios, this matrix of solid angles for intensity normalization suffers from a large loss of accuracy because of the neglect of the change in lateral size *dx*. In contrast, the rigorous Fraunhofer diffraction yields the analytic mapping relation for intensity normalization from the Eq. (11). In the meanwhile, the rigorous Fraunhofer diffraction can solve for the diffraction distribution in ES space for CDIs, and combines with the measured diffraction pattern interpolated to the ES space to solve straightforwardly the phase retrieval inverse problem directly on the ES space, instead of other approximate diffraction space. According to the two strategies for the Ewald sphere effect, we conducted a comparison experiment and the results are shown in Fig. S6. It is evident that the geometric corrections strategy in Fig. S6a for intensity normalization exhibits a discernible decline in imaging resolution in comparison to the rigorous Fraunhofer diffraction in Fig. S6b. From Figs. S6c and S6d, we can also see that the diffraction curvature of the corrected diffracted field is identical, with differences in pixel normalization intensities and diffraction propagation model for CDIs. Especially in Fig. S6f, the imaging resolution after the ES effect correction via the geometric corrections has degraded to 0.390 μm. However, in the same case of the rigorous Fraunhofer diffraction, the imaging resolution is 0.360 μm, close to the limit of 0.5 (*k* = 0.501) in imaging factor and successfully pushing the Abbe diffraction limit.


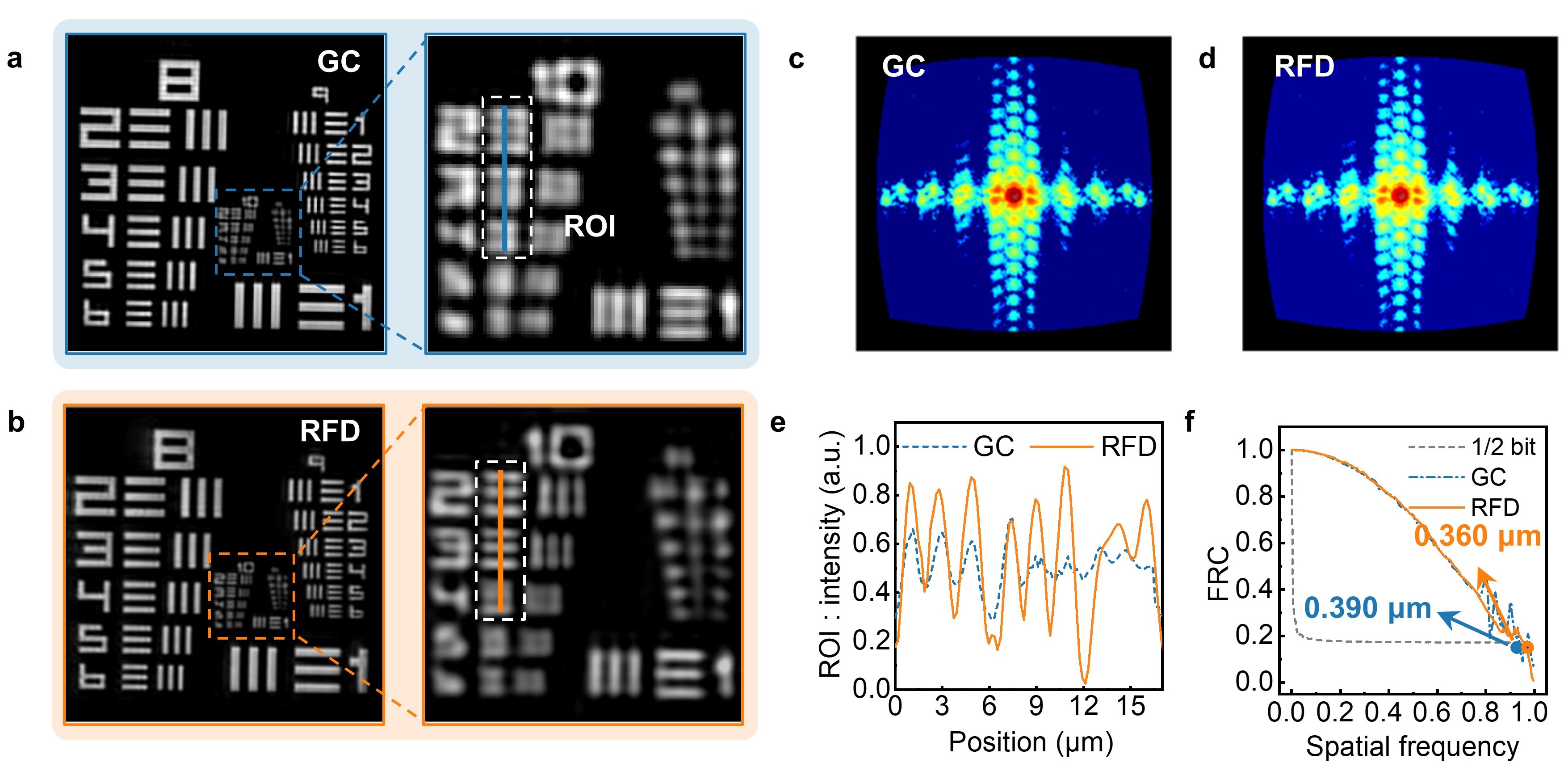


**Fig. S6 Comparison experiments for the ES effect**. **a, b,** Imaging results by the GC and the RFD, respectively. **c, d,** ES space diffraction fields by the GC and the RFD, respectively. **e,** Line traces. **f,** Fourier ring correlation.

## Reference

1. Chang, H., Enfedaque, P. & Marchesini, S. Blind ptychographic phase retrieval via convergent alternating direction method of multipliers. *SIAM Journal on Imaging Sciences* **12**, 153–85(2019).
2. Odstrčil, M., Menzel, A. & Guizar-Sicairos, M. Iterative least-squares solver for generalized maximum likelihood ptychography. *Optics Express* **26**, 3108-3123 (2018).
3. Tian, L., Li, X., Ramchandran, K. & Waller, L. Multiplexed coded illumination for Fourier Ptychography with an LED array microscope. *Biomedical Optics Express* **5**, 2376-2389 (2014).
4. Maiden, A., Johnson, D. & Li, P. Further improvements to the ptychographical iterative engine. *Optica* **4**, 736-745 (2017).
5. Chang, X., Jiang, S., Hu, Y., Zheng, G. & Bian, L. Pixel super-resolved lensless on-chip sensor with scattering multiplexing, *ACS Photonics* **10**, 2323–2331(2023).
6. Loetgering, L., Du, M., Eikema, K. S. E. & Witte, S. zPIE: an autofocusing algorithm for ptychography. *Optics Letter* **45**, 2030-2033 (2020).
7. Ruan, T., et al, Adaptive total variation based autofocusing strategy in ptychography. *Optics and Lasers in Engineering.*, **158**, 107136 (2022).
8. Zhang, F. et al. Translation position determination in ptychographic coherent diffraction imaging. *Optics Express* **21**, 13592-13606 (2013).
9. Liu, L. et al, An Efficient and Robust Self-calibration Algorithm for Translation Position Errors in Ptychography. *IEEE Transactions on Instrumentation and Measurement* **73**, 4503712 (2024).
10. Liu, L. et al. Resolution-Enhanced Lensless Ptychographic Microscope Based on Maximum-Likelihood High-Dynamic-Range Image Fusion. *IEEE Transactions on Instrumentation and Measurement* **73**, 4502711(2024).
11. Seaberg, M. D. et al. Ultrahigh 22 nm resolution coherent diffractive imaging using a desktop 13 nm high harmonic source. *Optics Express* **19**, 22470-22479 (2011).
12. Gardner, D. F. et al. High numerical aperture reflection mode coherent diffraction microscopy using off-axis apertured illumination. *Optics Express* **20**, 19050-19059 (2012).
13. Raines K. S. et al. Three-dimensional structure determination from a single *view*. *Nature* **463**, 214–217 (2010).
14. Zurch, M. W. *High-Resolution Extreme Ultraviolet Microscopy: Imaging of Artificial and Biological Specimens with Laser-Driven Ultrafast XUV Sources* (Springer, 2015).
